# Design of experiments for a confirmatory trial of precision medicine

**DOI:** 10.1016/j.jspi.2018.06.004

**Published:** 2019-03

**Authors:** Kim May Lee, James Wason

**Affiliations:** MRC Biostatistics Unit, School of Clinical Medicine, University of Cambridge, UK

**Keywords:** Design of experiments, Regression model, Treatment randomization scheme, Weighted L-optimality

## Abstract

Precision medicine, aka stratified/personalized medicine, is becoming more pronounced in the medical field due to advancement in computational ability to learn about patient genomic backgrounds. A biomaker, i.e. a type of biological process indicator, is often used in precision medicine to classify patient population into several subgroups. The aim of precision medicine is to tailor treatment regimes for different patient subgroups who suffer from the same disease. A multi-arm design could be conducted to explore the effect of treatment regimes on different biomarker subgroups. However, if treatments work only on certain subgroups, which is often the case, enrolling all patient subgroups in a confirmatory trial would increase the burden of a study. Having observed a phase II trial, we propose a design framework for finding an optimal design that could be implemented in a phase III study or a confirmatory trial. We consider two elements in our approach: Bayesian data analysis of observed data, and design of experiments. The first tool selects subgroups and treatments to be enrolled in the future trial whereas the second tool provides an optimal treatment randomization scheme for each selected/enrolled subgroups. Considering two independent treatments and two independent biomarkers, we illustrate our approach using simulation studies. We demonstrate efficiency gain, i.e. high probability of recommending truly effective treatments in the right subgroup, of the optimal design found by our framework over a randomized controlled trial and a biomarker–treatment linked trial.

## Highlights

• A classical randomized controlled trial fails to identify subgroup treatment effect.
• Standard enriched designs may miss out potential patient subgroups.
• A standard multi-arm design could be inefficient for a trial of precision medicine.
• A data-driven design framework could provide efficient designs for future trials.

## 1 Introduction

A randomized controlled trial (RCT) has been the gold standard for testing a new intervention in medicine, especially in phase III confirmatory studies. Many treatments work differently in different patient subgroups, and in this case RCTs which enroll all patients are not necessarily the most efficient approach in phase III. Instead enriched designs that recruit patients likely to benefit have considerable advantages. There is a danger that enriching a phase III trial too much may lead to missing out on a patient subgroup that would have actually benefited.

This therefore motivates phase II trials of targeted agents investigating not only whether a drug works but in which patient subgroups it works in. Several ‘biomarker-driven’ trial designs have been proposed to allow investigation of multiple treatment arms in different patient subgroups Buxton et al. (2014), Kaplan et al. (2013), Kaplan (2015), Middleton et al. (2015). In the case where each treatment can be tested in each subgroup, the number of hypotheses to be tested in a trial can be very large. Some recent papers providing an overview of biomarker-driven trial designs include Antoniou et al. (2016), Renfro and Sargent (2017), Antoniou et al. (2017) and Parmar et al. (2017).

One important aspect of biomarker-driven trial designs that has not been well researched is how to use the information collected from a phase II trial assessing multiple treatments and biomarkers to design the most efficient phase III designs. In particular it would be very useful to have a framework which determines which treatments should be tested in phase III, and in which biomarker subgroups. There has been some work in the context of evaluating a single experimental treatment (Ondra et al., 2016), but to our knowledge none that investigates novel multi-arm phase II biomarker-driven trial designs.

Considering a regression model with first order interaction terms, we propose a tool to design a confirmatory trial based on the analysis of an observed phase II trial or a historical study. There are two elements in this tool: Bayesian data analysis on data of a phase II trial, and the application of design of experiments to finding an optimal design for future experiment. The focus of our tool is to find an efficient design that could reject false null hypotheses in the confirmatory trial with high power.

Bayesian data analysis is a flexible approach where the knowledge and confidence of clinicians can be incorporated into the framework via the specification of a prior distribution. When the sample size of the observed trial is small, we suggest bootstrapping the data for the Bayesian analysis, and conjecture a subset of hypotheses that would be tested in a confirmatory trial based on a posterior predictive distribution from the analysis. We then use the notion of design of experiments to find the optimal treatment randomization scheme for the future experiments based on these information. Design of experiments is an approach that provides guidance on data collection such that sufficient information could be collected for a future experiment. We consider a weighted version of L-optimal criterion that resemble the idea of Morgan and Wang (2010) where they consider weighted D-, A-, and E-optimal designs for a factorial model. Sverdlov and Rosenberger (2013) review methods on finding optimal allocation for multi-arm clinical trials, where the design depends on the unknown parameters of a factorial model. We note that the Bayesian data analysis in our framework is independent of the commonly used Bayesian optimal design framework, see for example Kathryn and Verdinelli (1995) for the review on Bayesian optimal design framework. Our framework can be generalized to finding a Bayesian optimal design for generalized linear and nonlinear models.

The structure of the paper is as follows. We present a statistical model and hypothesis testing procedure for the trial with biomarker setting in Section 2. We introduce our novel design approach in Section 3, and conduct simulation study to compare the performance of the proposed optimal designs with two commonly employed designs in Section 4. We discuss our work and provide some insights into future research topics in Section 5.

### 1.1 Motivating trial

As the motivation for the work that follows, we consider a phase II trial that, at the time of writing, is under consideration for funding. This trial will test two experimental targeted treatments (T1 and T2), against chemotherapy control, for high grade serous ovarian cancer. Two biomarkers are included (B1 and B2) with it being thought likely (but not definite) that T1 will work best in B1 positive patients and T2 in B2 positive patients. Patients can be positive for B1, B2, both or neither.

The endpoint used for efficacy is six month change in the level of circulating tumor DNA in the blood, which will be treated as normally distributed on the log scale. The objective of the phase II trial is to determine which of T1 and T2 should be tested in a larger phase III trial, and in which patient subgroups. The methodology in this paper will be used for helping to make this decision.

## 2 Background and notation

Let vector $x_{i}=\left(x_{i1},\ldots ,x_{iL}\right)$ be a biomarker profile of patient i where $x_{il}=1$ represents patient i is positive for biomarker l, and $x_{il}=0$ otherwise, l=1,…,L; $T_{ik}$ be the experimental treatment indicator where $T_{ik}=1$ indicates that patient i receives treatment k. The response model for patient i is

$$y_{i}=\alpha +\overset{K}{\underset{k=1}{\sum }}T_{ik}\beta_{k}+\overset{L}{\underset{l=1}{\sum }}x_{il}\gamma_{l}+\overset{K}{\underset{k=1}{\sum }}\overset{L}{\underset{l=1}{\sum }}T_{ik}x_{il}\delta_{kl}+\epsilon_{i},$$

where α is the placebo/control effect for a patient with a negative biomarker profile, i.e. $x_{i}=\left(0,\ldots ,0\right)$, $\beta_{k}$ is the main effect of experimental treatment k, $\gamma_{l}$ is the main effect of biomarker l, and $\delta_{kl}$ is the interaction between treatment k and biomarker l. A placebo/control treatment is indicated by $T_{ik}=0$, ∀k=1,…,K. The residual errors, $\epsilon_{i}$, are assumed to be identically and independently distributed, and that they are normally distributed with zero mean and a common variance $\sigma^{2}$, i.e. $\epsilon_{i}\overset{iid}{∼}N\left(0,\sigma^{2}\right)$, i=1,…,n.

As an example, consider a trial where there are two experimental treatments and two biomarkers, i.e. K=2 and L=2, and that each patient receives only one treatment (either $T_{i1}=1$ or $T_{i2}=1$) or a placebo/control treatment, $T_{i1}=0$ and $T_{i2}=0$. The response model is

(1) $$y_{i}=\alpha +\beta_{1}T_{i1}+\beta_{2}T_{i2}+\gamma_{1}x_{i1}+\gamma_{2}x_{i2}+\delta_{11}x_{i1}T_{i1}+\delta_{12}x_{i2}T_{i1}+\delta_{21}x_{i1}T_{i2}+\delta_{22}x_{i2}T_{i2}+\epsilon_{i}$$

$$=f\left(x_{il},T_{ik}\right)\theta +\epsilon_{i},$$

where

$$f\left(x_{il},T_{ik}\right)=\left(1,\;T_{i1},\;T_{i2},\;x_{i1},\;x_{i2},\;x_{i1}T_{i1},\;x_{i2}T_{i1},\;x_{i1}T_{i2},\;x_{i2}T_{i2}\right)$$

is a row vector of the design matrix, denoted by X, of the regression model, and

$$\theta ={\left(\alpha ,\beta_{1},\beta_{2},\gamma_{1},\gamma_{2},\delta_{11},\delta_{12},\delta_{21},\delta_{22}\right)}^{T}$$

can be estimated by least squares estimator, $\overset{ˆ}{\theta }={\left(X^{T}X\right)}^{-1}X^{T}y$, that has $cov\left(\overset{ˆ}{\theta }\right)={\left(X^{T}X\right)}^{-1}\sigma^{2},$ where

$$X^{T}X=\overset{n}{\underset{i=1}{\sum }}f^{T}\left(x_{il},T_{ik}\right)f\left(x_{il},T_{ik}\right)=n\overset{m}{\underset{i^{\prime }=1}{\sum }}\frac{n_{i^{\prime }}}{n}f^{T}\left(x_{i^{\prime }},T_{i^{\prime }}\right)f\left(x_{i^{\prime }},T_{i^{\prime }}\right)=n\overset{m}{\underset{i^{\prime }=1}{\sum }}p_{i^{\prime }}f^{T}\left(x_{i^{\prime }},T_{i^{\prime }}\right)f\left(x_{i^{\prime }},T_{i^{\prime }}\right),$$

$\sum_{i^{\prime }=1}^{m}n_{i^{\prime }}=n$, $n_{i^{\prime }}$ and $p_{i^{\prime }}=\frac{n_{i^{\prime }}}{n}$ correspond to the number and proportion of patients who have the same $f^{T}\left(x_{i^{\prime }},T_{i^{\prime }}\right)$, and m denotes the number of unique $f^{T}\left(x_{i^{\prime }},T_{i^{\prime }}\right)$.

Without loss of generality, negative values indicate that the treatment is effective on the patients. We can test the existence of treatment effect on patients with different biomarker profiles by conducting hypothesis tests where

$$\begin{array}{cc} \text{null hypothesis:} & c_{r}^{\prime }\theta =0,\\ \text{alternative hypothesis:} & c_{r}^{\prime }\theta <0, \end{array}$$

$c_{r}$ is a vector that indicates the linear combination of regression parameters, i.e. the corresponding treatment effect that is different to the placebo/control, r=1,…,R is an index of the hypotheses that are possibly be tested in the study. As an example, consider patients who have biomarker profile $\left(x_{i1},x_{i2}\right)=\left(1,0\right)$, the difference between the effect of $T_{i1}=1$ and $T_{ik}=0$ ∀k on this subgroup is $\beta_{1}$ + $\delta_{11}$. Table 1 shows the treatment effect and the difference to the control/placebo effect for each subgroup where the responses follow (1), Table 2 shows the possible values of $c_{r}$ when model (1) is the analysis model.

**Table 1:** Biomarker–treatment combinations, model parameters for each combination, difference between the effect of a treatment and the placebo/control within the subgroups, and randomization scheme of some designs.

$i^{\prime }$	$x_{i1}$	$x_{i2}$	$T_{i1}$	$T_{i2}$	Treatment effect	Difference to control	$\xi_{rct}$	$\xi_{tlt}$	$\xi_{L}^{*}$
1	0	0	0	0	α		1/3	1/3	0.46
2	0	0	1	0	α + $\beta_{1}$	$\beta_{1}$	1/3	1/3	0.54
3	0	0	0	1	α + $\beta_{2}$	$\beta_{2}$	1/3	1/3	0.00

4	0	1	0	0	α + $\gamma_{2}$		1/3	1/2	0.46
5	0	1	1	0	α + $\gamma_{2}$ + $\beta_{1}$ + $\delta_{12}$	$\beta_{1}$ + $\delta_{12}$	1/3	0	0.00
6	0	1	0	1	α + $\gamma_{2}$ + $\beta_{2}$ + $\delta_{22}$	$\beta_{2}$ + $\delta_{22}$	1/3	1/2	0.54

7	1	0	0	0	α + $\gamma_{1}$		1/3	1/2	0.46
8	1	0	1	0	α + $\gamma_{1}$ + $\beta_{1}$ + $\delta_{11}$	$\beta_{1}$ + $\delta_{11}$	1/3	1/2	0.54
9	1	0	0	1	α + $\gamma_{1}$ + $\beta_{2}$ + $\delta_{21}$	$\beta_{2}$ + $\delta_{21}$	1/3	0	0.00

10	1	1	0	0	α + $\gamma_{1}$ + $\gamma_{2}$		1/3	1/3	0.47
11	1	1	1	0	α + $\gamma_{1}$ + $\gamma_{2}$ + $\beta_{1}$ + $\delta_{11}$ + $\delta_{12}$	$\beta_{1}$ + $\delta_{11}$ + $\delta_{12}$	1/3	1/3	0.00
12	1	1	0	1	α + $\gamma_{1}$ + $\gamma_{2}$ + $\beta_{2}$ + $\delta_{22}$ + $\delta_{21}$	$\beta_{2}$ + $\delta_{22}$ + $\delta_{21}$	1/3	1/3	0.53

$\xi_{rct}=$ randomized controlled trial; $\xi_{tlt}=$biomarker–treatment linked trial; $\xi_{L}^{*}=$optimal design for the first illustration.

We reject the null hypothesis, $c_{r}^{\prime }\theta =0$, if the test statistic,

$$\frac{c_{r}^{\prime }\overset{ˆ}{\theta }}{{\left[c_{r}^{\prime }\left[cov\left(\overset{ˆ}{\theta }\right)\right]c_{r}\right]}^{1∕2}}<-Z_{\alpha^{\prime }},$$

where $Z_{\alpha^{\prime }}$ is the $\left(1-\alpha^{\prime }\right)\text{\%}$ quantile of a standard normal distribution, with $\alpha^{\prime }$ Type 1 error. It is a desired property that $cov\left(\overset{ˆ}{\theta }\right)$ is small in statistical analysis, reflecting that the data provides good and sufficient information to understand the underlying random process. In terms of a hypothesis test, smaller values of $cov\left(\overset{ˆ}{\theta }\right)$ lead to higher probability to reject false null hypotheses (i.e. when the true value $c_{r}^{\prime }\theta <0$). We note that the real parameter values, θ, are not known in practice but the covariance matrix of the least squares estimator is inversely proportional to the information matrix, $X^{T}X$, which does not depend on θ.

**Table 2:** The possibly tested hypotheses when model (1) is the analysis model. The rth column represents the vector $c_{r}$.

r	1	2	3	4	5	6	7	8
θ	$c_{r}$							
α	0	0	0	0	0	0	0	0
$\beta_{1}$	1	1	1	1	0	0	0	0
$\beta_{2}$	0	0	0	0	1	1	1	1
$\gamma_{1}$	0	0	0	0	0	0	0	0
$\gamma_{2}$	0	0	0	0	0	0	0	0
$\delta_{11}$	0	1	0	1	0	0	0	0
$\delta_{12}$	0	0	1	1	0	0	0	0
$\delta_{21}$	0	0	0	0	0	1	0	1
$\delta_{22}$	0	0	0	0	0	0	1	1

## 3 Design of confirmatory trial

To investigate the effectiveness of treatments on biomarker subgroups, the biomarker profiles need to be known in advance. Conventional designs such as a randomized controlled trial would fail to study the treatment effect on different subgroups when the information on biomarker profiles is unavailable. On the other hand, a biomarker–treatment linked trial would not administer a linked-treatment to a subgroup with a negative biomarker, and administer all possible tested treatments to the subgroup who has all negative biomarkers. If treatments work only in biomarker positive subgroups, an enrichment design could be implemented where the subgroup with all negative biomarkers is excluded in the trial. All of these commonly used designs consider equal randomization probabilities to assign treatments within subgroups. The columns, $\xi_{rct}$ and $\xi_{tlt}$, in Table 1 show the randomization schemes of a randomized controlled trial and of a treatment linked trial for a study with two independent biomarkers and two independent treatments.

Instead of using these conventional designs, we propose to design a phase III/confirmatory trial based on an analysis of a phase II/exploratory study. The idea of our design framework is as follows: analyze an observed data using a Bayesian framework to provide guidance on selecting a subset of R possibly tested hypotheses and subgroups of patients to enroll in the confirmatory trial; formulate an analysis model for the future experiment and find an optimal randomization scheme. We propose to formulate an analysis model parsimoniously at the design stage to save costs on enrollment and data collection for the future confirmatory trial. For a chosen model, we consider the notion of design of experiments whereby the optimal treatment allocation scheme is of interest. An optimal design framework chooses the setting of a confirmatory experiment through the design matrix X such that some functions of $cov\left(\overset{ˆ}{\theta }\right)$ are minimized. The following sections illustrate the key ideas of our framework: investigate which hypotheses are more beneficial to focus on in the future experiment, and find an efficient design that has high probability of recommending truly effective treatments in the right subgroups.

### 3.1 Specification of relative importance of hypotheses

We now illustrate the selection of hypotheses and subgroups using the data of a previous experiment such as a phase II study. The idea is to consider an analysis approach that aims to provide insight into a predictive distribution of model parameters of the future confirmatory trial. We consider a Bayesian approach of data analysis to account for the unseen uncertainty when selecting a subset of hypotheses that will be tested in the confirmatory trial, reflecting the prior belief or confidence of an experimenter in terms of what might happen in the future experiment. This is achieved by specifying a prior distribution for the model parameters. When the sample size of an observed study is small, we propose to use simple bootstrap procedure on the data, and conduct the Bayesian analysis in each bootstrap replication to overcome the variability of using only one set of data to make selection of hypotheses. If only the summary statistics of the phase II trial are available, we recommend to replicate the phase II trial and explore the operating characteristics of the optimal designs for a future experiment, where each optimal design is obtained based on the analysis of a replication of the phase II trial.

To illustrate our framework, we use a conjugate prior in the following presentation. Markov chain Monte Carlo (MCMC) sampling could be used when the posterior distribution is intractable. We define the total sample size of the phase II trial as $n_{II}$, the vector of observed responses as $y_{o}$, design matrix as $X_{0}$, and regression parameters of the response model of phase II as $\theta_{II}$. For an observed study with a small $n_{II}$, we first bootstrap $y_{o}$ and $X_{0}$ with replacement and conduct Bayesian data analysis on each bootstrap sample. For example, using a normal-inverse-gamma prior, $NIG\left(\theta_{0},V_{0},a,b\right)$, for $\left(\theta_{II},\sigma^{2}\right)$, we obtain a posterior distribution $NIG(\theta_{m}^{*}$, $V^{*},a^{*},b^{*})$,

$$\theta_{m}^{*}={\left(V_{0}^{-1}+X_{0}^{T}X_{0}\right)}^{-1}\left(V_{0}^{-1}\theta_{0}+X^{T}y_{o}\right),V^{*}={\left(V_{0}^{-1}+X_{0}^{T}X_{0}\right)}^{-1},a^{*}=a+\frac{n_{II}}{2},b^{*}=b+\frac{1}{2}\left[\theta_{0}^{T}V_{0}^{-1}\theta_{0}+y_{o}^{T}y_{o}-{\left(\theta^{*}\right)}^{T}{\left(V^{*}\right)}^{-1}\theta^{*}\right]$$

for each bootstrapped sample. The marginal distribution of $\theta_{II}$ follows a multivariate t-distribution, $MVSt_{2a^{*}}\left(\theta_{m}^{*},\Sigma^{*}\right)$ with $\Sigma^{*}=\left(\frac{b^{*}}{a^{*}}\right)V^{*}$ and $2a^{*}$ degree of freedom.

Let $w_{r}$ denote the relative importance of hypothesis r, r=1,…,R. We propose to generate large samples from the posterior distribution (use MCMC sampling for intractable posterior distribution) to compute

$$P_{r}=P\left(c_{r}^{\prime }\theta <\tau_{r}\right)$$

where $c_{r}^{\prime }\theta$ is approximately normally distributed with mean $c_{r}^{\prime }\theta_{m}^{*}$, and variance $c_{r}^{\prime }\Sigma^{*}c_{r}$, and $\tau_{r}$ could be the minimum uninteresting treatment difference threshold for hypothesis r. We then find $E\left(P_{r}\right)$ where the expectation is averaged across the number of bootstrap replications, and compute $w_{r}$, the relative importance of hypothesis r, by

$$w_{r}=\left\{\begin{array}{cc} E\left(P_{r}\right)\; & \text{if }E\left(P_{r}\right)\geq \kappa \text{, where }\kappa \text{ is a user-specified threshold;}\\ 0\; & \text{otherwise.} \end{array}\right)$$

Hypothesis r is selected and the corresponding subgroup is enrolled into the confirmatory trial if $w_{r}>0$. These information is then used to formulate a parsimonious linear regression model and the design criterion for finding an optimal randomization scheme for the future confirmatory trial. Note that $\sum_{r=1}^{R}w_{r}$ need not sum up to 1 in the design problem.

### 3.2 Design of experiments

We now describe the design framework for finding an optimal design. After formulating the analysis model parsimoniously for the future experiment such that the parameters of interest are estimable, we want to find an optimal design,

$$\xi^{*}=\left\{\begin{array}{ccc} \left(x_{1},T_{1}\right) & \cdots & \left(x_{m},T_{m}\right)\\ p_{1} & \cdots & p_{m} \end{array}\right\},$$

that minimizes

(2) $$\overset{R}{\underset{r=1}{\sum }}w_{r}\;c_{r}^{\prime }\left[cov\left(\overset{ˆ}{\theta }\right)\right]c_{r}\propto \overset{R}{\underset{r=1}{\sum }}w_{r}\;c_{r}^{\prime }{\left(n\overset{m}{\underset{i^{\prime }=1}{\sum }}p_{i^{\prime }}f^{T}\left(x_{i^{\prime }l},T_{i^{\prime }k}\right)f\left(x_{i^{\prime }l},T_{i^{\prime }k}\right)\right)}^{-1}c_{r},$$

where $w_{r}\geq 0$ reflects the relative importance of the corresponding hypothesis r. This setup has several well-known optimality criteria as special cases: the design criterion is called L-optimality when $w_{r}=1\;\forall r$ (page 111 in Luc Pronzato and Andrej Pazman (2013)); it is an A-optimality when the summation sums the individual variances of the model parameters and is a c-optimality when R=1 and $w_{1}=1$. The incorporation of $w_{r}$ into the standard L-optimality resembles the idea of Morgan and Wang (2010) where the authors consider weighted D-, A-, and E-optimal designs for a factorial model.

A design ξ is called a continuous design when $np_{i^{\prime }}$ need not be a positive integer in the optimization search but $\sum_{i^{\prime }=1}^{m}p_{i^{\prime }}=1$, $p_{i^{\prime }}\geq 0$. Otherwise ξ is called an exact design where $np_{i^{\prime }}$ is a positive integer. The notion of a continuous design facilitates the search of an optimal design over a design region. A rounding procedure can be applied to the continuous design for practical implementation, see for example Pukelsheim and Rieder (1992). The interpretation of $p_{i^{\prime }}$ in the conventional design framework context is that it is the suggested proportion of subgroups who have the corresponding $\left(x_{i^{\prime }},T_{i^{\prime }}\right)$. In the trial with biomarker setting, it is difficult to enroll the patient subgroups in practice according to the exact proportions. Hence, we use $p_{i^{\prime }}$ to compute the randomization probability for each selected subgroup instead. Note that no rounding procedure is needed in this case. For a given total sample size, we find $p_{i^{\prime }}$ of $\xi^{*}$ by minimizing (2), subject to $\sum_{i^{\prime }=1}^{m}p_{i^{\prime }}=1$. The relative importance of the selected subset of hypotheses and the subgroups are reflected by the values of $w_{r}$, r=1,…,R, and biomarker–treatment combination $\left(x_{i^{\prime }},T_{i^{\prime }}\right),i^{\prime }=1,\ldots ,m$. To avoid confusion, we use the same index r to denote the hypotheses in the design framework even some of the hypotheses are not selected to be tested in the future experiment.

As an example, consider that we are interested in testing only the following null hypotheses:

1. $\beta_{1}=0$; enroll biomarker–treatment combinations, $\left(x_{i1},x_{i2},T_{i1},T_{i2}\right)=\left(0,0,0,0\right)$ and (0,0,1,0).
2. $\beta_{1}+\delta_{11}=0$; enroll $\left(x_{i1},x_{i2},T_{i1},T_{i2}\right)=\left(1,0,0,0\right)$ and (1,0,1,0).
3. $\beta_{2}+\delta_{22}=0$; enroll $\left(x_{i1},x_{i2},T_{i1},T_{i2}\right)=\left(0,1,0,0\right)$ and (0,1,0,1).
4. $\beta_{2}+\delta_{22}+\delta_{21}=0$; enroll $\left(x_{i1},x_{i2},T_{i1},T_{i2}\right)=\left(1,1,0,0\right)$ and (1,1,0,1).

We propose to employ

$$y_{i}=\alpha +\beta_{1}T_{i1}+\beta_{2}^{\prime }T_{i2}+\gamma_{1}x_{i1}+\gamma_{2}x_{i2}+\delta_{11}x_{i1}T_{i1}+\delta_{21}x_{i1}T_{i2}+\epsilon_{i},$$

where $\beta_{2}^{\prime }=\beta_{2}$ + $\delta_{22}$ instead of model (1) for the analysis of the future trial. An optimal design problem is to minimize

$$w_{1}cov\left({\overset{ˆ}{\beta }}_{1}\right)+w_{2}cov\left({\overset{ˆ}{\beta }}_{1}+{\overset{ˆ}{\delta }}_{11}\right)+w_{7}cov\left({\overset{ˆ}{\beta }}_{2}+{\overset{ˆ}{\delta }}_{22}\right)+w_{8}cov\left({\overset{ˆ}{\beta }}_{2}+{\overset{ˆ}{\delta }}_{22}+{\overset{ˆ}{\delta }}_{21}\right),$$

subject to $\sum_{i^{\prime }=1}^{12}p_{i^{\prime }}=1$, $p_{i^{\prime }}=0$ for $i^{\prime }=3,5,9,11$. We note that the parameter $\delta_{12}$ would not be estimable when the optimal design is used because none of the selected patients would have $x_{i2}T_{i1}=1$. Besides that, we can only estimate the combined effect of $\beta_{2}+\delta_{22}$ here as the enrolled patients would have $T_{i2}=1$ only if $x_{i2}=1$. Note that if the experimenter chooses κ=0 and hence $w_{r}>0\;\forall r$, model (1) would be used as if in the randomized controlled trial where all subgroups are enrolled into the trial.

In general, the analysis model for a future experiment could be formulated by considering the model parameters of the selected hypotheses and the subgroup biomarker profiles. A linear combination of model parameters might be replaced by a new variable accordingly such that the information matrix is of full rank.

## 4 Illustration: a trial with two biomarkers and two treatments

In this section, we conduct simulation studies to illustrate the application of our framework to finding an optimal design for a confirmatory trial based on Bayesian analysis of a historical study, and make comparisons on the operating characteristics of a randomized controlled trial, $\xi_{rct}$, a biomarker–treatment linked trial, $\xi_{tlt}$, and optimal designs. Throughout the illustration, we consider the presence of two independent biomarkers with prevalence rate of 0.3 each, and two independent treatments that are linked to the biomarkers. In the first part of this illustration, we simulate a set of phase II data for finding an optimal design based on our framework, and use the bootstrap estimates as the true model parameters in the simulation of a confirmatory trial. We replicate a confirmatory trial using the randomization scheme of the designs to study the performance of different designs. In the second part of the illustration, we do not bootstrap a set of data but replicate the phase II trial according to a set of model parameters to explore the operating characteristics of different optimal designs, where each optimal design is found based on the analysis of a replicated phase II study. We compare the performance of these optimal designs with $\xi_{rct}$ and $\xi_{tlt}$ in the simulation of a confirmatory trial where the true model parameters are the same as those used in the replication of the phase II trial. The first illustration shows the role of κ>0 on the optimal design when a set of phase II data is available; the latter illustration reflects the situation where only the summary statistics of a phase II trial are available, and shows the role of $w_{r}$ on the optimal design when κ=0.

Consider a phase II randomized controlled trial, $\xi_{rct}$, that studies the effect of two independent treatments, $T_{i1}$ and $T_{i2}$, on patients where the information of two independent biomarkers, $x_{i1}$ and $x_{i2}$, are available. For $n_{II}=400$, we simulate biomarker profiles using binomial distributions with prevalence rate of 0.3 for each biomarker, treatment allocation with equal randomization probability, and a set of $y_{o}$ according to model (1) with $\epsilon_{i}\overset{iid}{∼}N\left(0,\sigma^{2}\right)$, $\sigma^{2}=1.15$ and $\theta_{II}={\left(-0.356,-0.213,0.240,1,1,-0.182,0.240,0.527,-0.586\right)}^{T}$. We note that model (1) has r=1,…,8, possible hypotheses (see Table 2). We choose prior values a=0.0001,b=0.0001, $\theta_{0}={\left(0,0,0,0,0,-0.1,0,0,-0.1\right)}^{T}$, and $V_{0}$ a diagonal matrix with $diag\left(V_{0}\right)=\left(1,1,1,1,1,2,1,1,2\right)$. The values of $\theta_{0}$ and $V_{0}$ are chosen to reflect the belief that the model parameters of a confirmatory experiment, apart from $\delta_{11}$ and $\delta_{22}$, have no effect on the responses. The variances of $\delta_{11}$ and $\delta_{22}$ are relatively larger than the variances of other parameters show that there are more uncertainty in the belief that $\delta_{11}$ and $\delta_{22}$ may have negative effect on the responses. We consider bootstrapping the data 10 000 times in this illustration. For each bootstrap sample, we compute $\theta_{m}^{*}$ and $\Sigma^{*}$, and generate 10 000 samples from the posterior distribution, $MVSt_{2a^{*}}\left(\theta_{m}^{*},\Sigma^{*}\right)$, to compute $P_{r}$ by choosing $\tau_{r}=0\;\forall r$. We then use all $P_{r}$ from the bootstrap replications to compute $E\left(P_{r}\right)$ and $w_{r}$ by choosing κ=0.5. Based on the values of $w_{r}$, we formulate a model parsimoniously and find an optimal design by minimizing (2) using the function *fmincon* in Matlab. In this illustration, we obtained $E\left(P_{1}\right)=0.761$, $E\left(P_{2}\right)=0.683$, $E\left(P_{3}\right)=0.326$, $E\left(P_{4}\right)=0.367$, $E\left(P_{5}\right)=0.097$, $E\left(P_{6}\right)=0.032$, $E\left(P_{7}\right)=0.883$ and $E\left(P_{8}\right)=0.501$. Hence, we have $w_{r}=E\left(P_{r}\right)$ for r=1,2,7,8, and $w_{r}=0$ for r=3,4,5,6, in this example. An optimal design denoted by $\xi_{L}^{*}$ is obtained for this setting.

To study the operating characteristics of the design, we conduct simulation study with a larger sample size to reflect the practice of a confirmatory trial. We compare the performance of $\xi_{L}^{*}$, with a randomized controlled trial, $\xi_{rct}$, and a biomarker–treatment linked trial, $\xi_{tlt}$. These designs are different in terms of number of subgroups and treatment allocation scheme, see Table 1. We use the same prevalence rate, i.e. 0.3 for both independent biomarkers, and sample size of 1000 in the simulation of the confirmatory trial, whereby the treatment randomization scheme follows a design. In each simulation, we replicate the biomarker profiles 100 times; for each replication of biomarker profiles, we replicate treatment allocation according to the randomization scheme 100 times. The responses are generated according to model (1) with the expected model parameters from the bootstrap replications, θ=(−0.326,−0.155,0.324,0.794,1.061,−0.014,0.426,0.290,−0.784), and $\sigma^{2}=1.15$. Using the simulated responses, we estimate the model parameters by least squares estimation, and compute the test statistics for hypothesis test with $\alpha^{\prime }=0.05$ in each replication. The number of times that a null hypothesis is rejected is averaged across the replications (both the replication of treatment allocation and biomarker profiles), giving the power of rejecting a null hypothesis if it is false, or a Type 1 error if it is true. In this illustration, we know $c_{r}^{\prime }\theta <0$, for r=1,2,7,8. We compute the expected number of correct rejection of false null hypotheses (ENCR) to make comparisons between the designs.

Table 3 shows the effect sizes from different hypotheses and the operating characteristics of different designs in the simulation of a confirmatory trial. In this illustration, we find that the power of rejecting $c_{7}^{\prime }\overset{ˆ}{\theta }$ is the largest whereas the power of rejecting $c_{8}^{\prime }\overset{ˆ}{\theta }$ is the smallest; the Type 1 error of rejecting $c_{5}^{\prime }\overset{ˆ}{\theta }$ and $c_{6}^{\prime }\overset{ˆ}{\theta }$ are close to 0. This shows that the true values of hypotheses play a major part in hypothesis test where large magnitude of model parameters is easier to detect than others that are close to the bound chosen for a hypothesis test. The other reason that the power of rejecting $c_{8}^{\prime }\overset{ˆ}{\theta }$ is so small is due to small subgroup sample size and the fact that the power of rejecting a null hypothesis with an interaction term is generally lower than that with only main effect parameters (Follmann, 2003). The expected sample size of this subgroup is 1000∗0.3∗0.3=90, which is not sufficient for detecting the treatment effect that is very close to zero. Comparing the designs, the optimal design generally achieves larger power of rejecting the false null hypotheses. Consider the expected number of correct rejection of false null hypotheses (ENCR), we find that $\xi_{rct}$ and $\xi_{tlt}$ would lose about 11% and 7% efficiency over $\xi_{L}^{*}$. Looking at testing $c_{1}^{\prime }\theta =0$, we find that the power could be increased significantly if the optimal design is used in the experiment, i.e. 0.11 and 0.12 more than what $\xi_{rct}$ and $\xi_{tlt}$ could achieve. The randomization scheme of the optimal design for this corresponding subgroup is 0.46 for receiving placebo and 0.54 for receiving treatment 1, whereas the later designs are having 1/3 for receiving placebo, treatment 1 and treatment 2. This shows that enrolling a less informative biomarker–treatment combination could be waste of resources. In particular, $c_{5}^{\prime }\theta =\beta_{2}$ is not significant in this illustration and that having the biomarker–treatment combination $i^{\prime }=3$ (see Table 1) in the experiment is not beneficial. Conjecturing which hypothesis is significant and to be tested in future experiment is a crucial step in designing the trial.

**Fig. 1:**
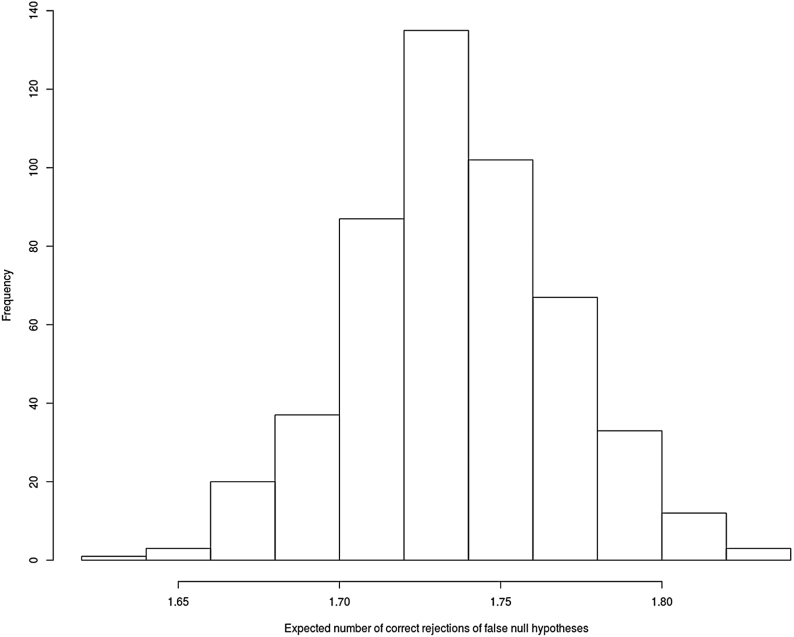
Frequency of optimal designs that achieve the expected number of correct rejection of false null hypotheses (ENCR).

 To illustrate further, we consider the same trial setting but that only the summary statistics of a phase II trial are available. We consider $c_{r}^{\prime }\theta =-0.2$ for r=1,2,7,8 and $c_{r}^{\prime }\theta >0$ for r=3,4,5,6, and $n_{II}=300$. Instead of bootstrapping one data set (that gives different parameter estimates in each bootstrap replication), we replicate a phase II trial 500 times according to model (1) with an equal probability randomization scheme. These replications reflect the variability of the error term and the randomization scheme while $c_{r}^{\prime }\theta$ remain the same across all the phase II replications. For each simulated phase II trial, we draw 10 000 samples from the posterior predictive distribution (i.e. $MVSt_{2a^{*}}\left(\theta_{m}^{*},\Sigma^{*}\right)$ that has the same prior as the previous illustration) and compute $w_{r}=E\left(P_{r}\right)$ by setting κ=0. An optimal design is then found based on the analysis of each phase II replication. Fig. 1 shows ENCR on the x-axis, that are achieved by the 500 optimal designs where each optimal design corresponds to a simulated phase II study. With total sample size of 1000, nominal Type 1 error of 5%, and true treatment difference of −0.2 for each hypothesis r=1,2,7,8, we find that the optimal designs are expected to reject at least 1.65 false null hypotheses in the simulation, whereas $\xi_{rct}$ and $\xi_{tlt}$ are giving 1.647 and 1.707 respectively.

**Table 3:** Effect sizes from different hypotheses and probability of rejecting a null hypothesis in the simulation of different designs.

Effect sizes from different hypotheses in the simulation of a confirmatory trial									
	$c_{1}^{\prime }\theta$	$c_{2}^{\prime }\theta$	$c_{3}^{\prime }\theta$	$c_{4}^{\prime }\theta$	$c_{5}^{\prime }\theta$	$c_{6}^{\prime }\theta$	$c_{7}^{\prime }\theta$	$c_{8}^{\prime }\theta$	
	−0.155	−0.169	0.135	0.121	0.324	0.750	−0.460	−0.034	

Probability of rejecting a null hypothesis in the simulation of a confirmatory trial									

	$c_{1}^{\prime }\overset{ˆ}{\theta }=0$	$c_{2}^{\prime }\overset{ˆ}{\theta }=0$	$c_{3}^{\prime }\overset{ˆ}{\theta }=0$	$c_{4}^{\prime }\overset{ˆ}{\theta }=0$	$c_{5}^{\prime }\overset{ˆ}{\theta }=0$	$c_{6}^{\prime }\overset{ˆ}{\theta }=0$	$c_{7}^{\prime }\overset{ˆ}{\theta }=0$	$c_{8}^{\prime }\overset{ˆ}{\theta }=0$	ENCR

$\xi_{rct}$	0.43	0.31	0.01	0.02	0.00	0.00	0.90	0.09	1.74
$\xi_{tlt}$	0.41	0.37	0.02	0.02	0.00	0.00	0.96	0.08	1.81
$\xi_{L}^{*}$	0.54	0.37	NA	NA	NA	NA	0.96	0.08	1.95

ENCR= expected number of correct rejection of false null hypotheses.

Table 4 shows the probability of rejecting each null hypothesis $c_{r}^{\prime }\theta =0$, r=1,…,8, achieved by $\xi_{best}^{*}$, $\xi_{worst}^{*}$, $\xi_{rct}$ and $\xi_{tlt}$. The former two are optimal designs that have the highest and the lowest ENCR (in the simulation of a confirmatory trial) out of the 500 optimal designs, where each design corresponds to a replication of a phase II study. Across all designs that have different randomization schemes, we find that the Type 1 error of rejecting each true hypothesis is no greater than 0.03 when the nominal $\alpha^{\prime }=0.05$ is used in the simulation. Comparing the four false null hypotheses that have the same true value of −0.2, we find that the power of rejecting $c_{1}^{\prime }\theta =0$ is the highest and $c_{8}^{\prime }\theta =0$ is the lowest across all designs. This is not surprising as the subgroups have different sample sizes and that with prevalence rate of 0.3 for both biomarkers, the total sample size of $i^{\prime }=1,2,3$, is expected to be 1000∗(1−0.3)∗(1−0.3)=490, and that of $i^{\prime }=10,11,12$, is 1000∗0.3∗0.3=90. The last column of Table 4 shows that $\xi_{rct}$ and $\xi_{tlt}$ would lose about 11% and 7% efficiency in terms of ENCR when compared with $\xi_{best}^{*}$. We find that $\xi_{worst}^{*}$ achieved a similar ENRC when compared to $\xi_{rct}$, but not better than $\xi_{tlt}$. The latter finding is mainly due to the fact that $\xi_{tlt}$ excluded $i^{\prime }=\text{5,9}$ (see Table 1) in the design whereas $\xi_{worst}^{*}$ enrolled these biomarker–treatment combinations in the trial, when $i^{\prime }=\text{5,9}$ have limited contribution on testing false null hypotheses r=1,2,7,8. The former result might be due to the random error of that particular replication of a phase II trial. If we bootstrap this replicated data assuming that they are the observed phase II data, we would then proceed as in the previous illustration but with κ=0, and could potentially obtain a better design than $\xi_{worst}^{*}$ and $\xi_{rct}$ for the future experiment.

**Table 4:** Probability of rejecting a null hypothesis. We know $c_{r}^{\prime }\theta <0$, r=1,2,7,8, and $c_{r}^{\prime }\theta >0$, r=3,4,5,6. Each row corresponds to the performance of a design.

	$c_{1}^{\prime }\overset{ˆ}{\theta }=0$	$c_{2}^{\prime }\overset{ˆ}{\theta }=0$	$c_{3}^{\prime }\overset{ˆ}{\theta }=0$	$c_{4}^{\prime }\overset{ˆ}{\theta }=0$	$c_{5}^{\prime }\overset{ˆ}{\theta }=0$	$c_{6}^{\prime }\overset{ˆ}{\theta }=0$	$c_{7}^{\prime }\overset{ˆ}{\theta }=0$	$c_{8}^{\prime }\overset{ˆ}{\theta }=0$	ENCR
$\xi_{best}^{*}$	0.67	0.42	0.02	0.02	0.01	0.02	0.43	0.32	1.83
$\xi_{worst}^{*}$	0.61	0.39	0.01	0.02	0.01	0.02	0.35	0.28	1.64
$\xi_{rct}$	0.59	0.39	0.02	0.02	0.01	0.02	0.38	0.29	1.65
$\xi_{tlt}$	0.56	0.45	0.03	0.03	0.01	0.03	0.45	0.24	1.71

## 5 Discussion

We have proposed a framework for designing confirmatory trials based on the analysis of a phase II study testing multiple experimental treatments in different biomarker subgroups. The design framework provides guidance on selecting biomarker–treatment combinations and a treatment randomization scheme.

When using a single regression model to analyze a multi-arm trial, the dimension of model parameters depends on the number of biomarker–treatment combinations. For example, in the presence of L binary biomarkers and K experimental treatments, there are $2^{L}\times K$ possible hypotheses that test treatment differences between treatments and a placebo. To find an efficient design, we propose a framework to select a subset of hypotheses out of the many possibly tested hypotheses based on Bayesian data analysis of a phase II/historical study, and use the notion of design of experiments to find the treatment randomization scheme for implementing a future experiment. We show that doing a traditional randomized trial enrolling all patient subgroups is not the most efficient approach when the treatments work only on certain subgroups.

We propose selecting the hypotheses to be tested in a confirmatory trial based on Bayesian data analysis of the phase II study. When the sample size of the observed study is small, we suggest to consider bootstrapping the available data to minimize the bias in estimation (due to small sample) that may cause bias in hypothesis selection. When only summary statistics are available, we suggest simulating the phase II study and exploring the operating characteristics of optimal designs before choosing one design for implementation. The above illustration shows that $w_{r}$, κ and τ play important roles in designing an experiment. In practice, κ and τ should be chosen carefully in discussion with clinicians based on their experiences. We note that our framework is flexible that each hypothesis may have different κ and τ. The specification of a prior distribution is another aspect that should reflect the knowledge of the clinicians on the likely magnitude of the treatment effect. When the posterior predictive distribution of model parameters is not in closed-form, we suggest to draw large samples using MCMC approaches, aiming to approximate the distribution of future model parameters by normal approximation.

Concerning the values of $w_{r}$, we find that small aberration on each $w_{r}$ does not affect the optimal randomization scheme notably. The operating characteristics of a design depend on the true model parameters more than $w_{r}$. However, the true model parameters are never known in practice. Instead of enrolling subgroups according to the optimal proportion, $p_{i}$, i=1,…,m, as suggested by the classical design framework, we convert them into randomization probability as it facilitates the implementation of a trial that considers subgroup stratification. However, this approach would complicate the sample size calculation especially when the prevalence rates of biomarkers are at the extreme end of the range. We consider using a single model in the analysis such that subgroups with small sample sizes may borrow information from other subgroups that have larger sample sizes.

We have presented the framework for normally distributed responses. We note that our framework could be extended to nonlinear models where adjustments to the Bayesian data analysis, computation of $w_{r}$, and the design criterion would be required. When the model is nonlinear, the covariance matrix of the parameters may depend on the unknown parameters, leading to issues with finding an optimal design. For a chosen value of the model parameters, the classical optimal design framework could provide a locally optimal design. An alternative to this is to use a Bayesian optimal design framework whereby a prior distribution of the model parameters is incorporated into the design criterion for finding an optimal design. We note that the proposed framework is different to the commonly known Bayesian optimal design framework as we only use historical data to estimate $w_{r}$ prior to finding an optimal design. Nevertheless, our framework could be extended to the nonlinear situation accordingly. For example, the observed phase II data may be used to construct the prior distribution for the unknown parameters in the Bayesian design framework, while the prior distribution in the Bayesian analysis of phase II data reflects the prior knowledge or confidence of the clinicians on the future experiment. These two different prior distributions could be the same or different. Other possible extensions of our framework could be the incorporation of missing data, see Lee et al. (2017a), Lee et al. (2017b), and incorporation of cost functions, see Cook and Fedorov (1995).

One of the potential topics for future research is to account for the population drift or change in baseline measure of subgroups in the design framework. When data of control groups from several small trials that studied the same disease are available, having a design framework that considers this aspect could be beneficial for the clinical community. See for example Thall and Simon (1990) and Boonstra et al. (2016) who consider the design and information gain when historical control data is incorporated, and van Rosmalen et al. (2017) for the review of methods for incorporating historical control data. Besides that, we have not accounted for all the aspects of an analysis plan. For example, when a single statistical model is chosen to analyze all data, most of the hypotheses are not independent as the information from different subgroups is shared across themselves. Future work could focus on constructing optimal designs that account for issues such as multiplicity while optimizing the hypotheses selection as our design framework has done here. The issue that arose in subgroup sample size calculations may also be incorporated and addressed in the prospective design framework.

To conclude, we have proposed a novel design framework for designing a biomarker driven confirmatory trial based on the analysis of an observed experiment which provides an increased chance of determining which subgroups a targeted treatment genuinely works in.
